# Metabolite profiling of yam (*Dioscorea spp*.) accessions for use in crop improvement programmes

**DOI:** 10.1007/s11306-017-1279-7

**Published:** 2017-10-14

**Authors:** Elliott J. Price, Ranjana Bhattacharjee, Antonio Lopez-Montes, Paul D. Fraser

**Affiliations:** 1 Royal Holloway University of London, Egham, Surrey, TW20 0EX UK; 2 Royal Botanic Gardens, Kew, Richmond, Surrey, TW20 3AB UK; 3 International Institute of Tropical Agriculture, Oyo Road, PMB 5320 Ibadan, Nigeria

**Keywords:** Yam, *Dioscorea*, Metabolomics, Crop breeding, Natural variation

## Abstract

**Introduction:**

Ninety-seven percent of yam (*Dioscorea* spp.) production takes place in low income food deficit countries (LIFDCs) and the crop provides 200 calories a day to approximately 300 million people. Therefore, yams are vital for food security. Yams have high-yield potential and high market value potential yet current breeding of yam is hindered by a lack of genomic information and genetic resources. New tools are needed to modernise breeding strategies and unlock the potential of yam to improve livelihood in LIFDCs.

**Objectives:**

Metabolomic screening has been undertaken on a diverse panel of *Dioscorea* accessions to assess the utility of the approach for advancing breeding strategies in this understudied crop.

**Methods:**

Polar and lipophilic extracts from tubers of accessions from the global yam breeding program have been comprehensively profiled via gas chromatography-mass spectrometry.

**Results:**

A visual pathway representation of the measured yam tuber metabolome has been delivered as a resource for biochemical evaluation of yam germplasm. Over 200 compounds were routinely measured in tubers, providing a major advance for the chemo-typing of this crop. Core biochemical redundancy concealed trends that were only elucidated following detailed mining of global metabolomics data. Combined analysis on leaf and tuber material identified a subset of metabolites which allow accurate species classification and highlighted the potential of predicting tuber composition from leaf profiles. Metabolic variation was accession-specific and often localised to compound classes, which will aid trait-targeting for metabolite markers.

**Conclusions:**

Metabolomics provides a standalone platform with potential to deliver near-future crop gains for yam. The approach compliments the genetic advancements currently underway and integration with other ‘–omics’ studies will deliver a significant advancement to yam breeding strategies.

**Electronic supplementary material:**

The online version of this article (doi:10.1007/s11306-017-1279-7) contains supplementary material, which is available to authorized users.

## Significance statement

The tubers of numerous *Dioscorea* (yam) species are an important staple of many tropical countries yet; numerous constraints hinder breeding, including a lack of genetic resources. Biochemical phenotyping accessions of the breeding program can be rapidly implemented to advance yam breeding strategies. The present study illustrates utility of metabolomics as a useful tool and resource to augment the breeding of new yam varieties with improved consumer and agronomic traits.

## Introduction

Over the last two decades global yam production has doubled, reaching a total production of 60 million tonnes and having a net value internationally of ~$12 billion. Approximately 97% of production is from low income food deficit countries (LIFDCs) (Food and Agriculture Organization of the United Nations 2015), with Western Africa accounting for over 90% of total production each year in this period. In spite of high total production, no Western African country is in the top five countries for yield of this crop per area farmed (Food and Agriculture Organization of the United Nations n.d.).

Compared to cassava, yam is four times more expensive per calorie to produce (Diop 1998), with increased labour requirements (Nweke and Ezumah 1992) and lower yields (Kenyon et al. 2006). Despite these comparative parameters, in regions of low-technology farming, yams produce the highest amount of food per hectare and per season (Kumar 2014). Unlike other root and tuber crops, yams may be stored for a period of 4–6 months at ambient temperature (Bricas and Attaie 1997; Knoth 1993) and as such, yams are vital for the food security of growing regions. To date yams as a food source and cash crop have been understudied and underutilised (Mignouna et al. 2009; Nkamleu et al. 2009). This is surprising considering their high yield potential, high food and market values and numerous post-harvest options for storage and utilisation (Asiedu and Sartie 2010). These are all properties suited to the combating of food insecurity, achieving the United Nations Sustainable Development Goals and the sustainable intensification of agriculture.

Large species diversity confers a wide range of ecological and agronomic traits within the genus, providing intrinsic opportunity for crop improvements through the exploitation of natural variation (Fernie et al. 2006). Conventional breeding strategies have had significant successes (Mignouna et al. 2003) however, to create the step-change required to combat our ever increasing global population and diminishing resources, modern tools and resources must be developed and implemented. A lack of availability of next generation ‘–omics’ resources and information has hindered application of molecular breeding in yam (Bhattacharjee et al. 2011).

In order to fully exploit the potential of this crop species, resources and tools are required that add value to existing and new discovery pipelines. Adding value to these pipelines will result in better competitive varieties with improved and new traits, as well as increasing the rapidity with which the varieties can be generated. Recent advances in the application of next generation sequencing has enabled capture of genomes from non-model species, including yams, with the development of reference genomes for *D. rotundata* (Tamiru et al. 2017), *D. alata & D. dumetorum* and large-scale screening of genetic diversity via genotyping-by-sequencing (GBS) and whole genome re-sequencing (WGRS) of these species and crop wild relatives to allow trait mapping and identification of single nucleotide polymorphism (SNP) variants (Fukushima et al. 2009).

Genomics represents one level of cellular regulation when coupled with other ‘–omics’ technologies such as transcriptomics, proteomics, metabolomics and phenomics the power of trait characterisation is greatly improved (Fukushima et al. 2009). This is particularly true of metabolomics where traits of interest can, in many cases be directly associated with metabolite composition (Bino et al. 2004).

The quality traits of yams are understudied with regards to breeding, largely due to the complicated genetic architecture of *Dioscorea* hindering association mapping. Progression has been slow, even when implementing next-generation technologies, due to the lack of fundamental molecular knowledge. For example, the ploidy status of many accessions in current breeding programs is unknown. Only recently have the chromosome numbers of major edible species been clarified and definitive studies assessing ploidy have required inference through marker segregation (Arnau et al. 2009; Bousalem et al. 2006; Scarcelli et al. 2005). Furthermore, dioecy, intra and inter-species hybridisation and the large phenotypic plasticity of yams has made the scoring and association of numerous interdependent traits a convoluted process.

Metabolomics platforms produce large-scale biochemical phenotypes that can be representative of quality traits. Association of phenotype to metabolite profiles can provide measureable markers comparable to genetic quantitative trait loci (QTL) e.g. metabolite quantitative trait loci (mQTL) which can be recorded through breeding program both independent of underlying genetic mechanisms and combined with typical genomic analysis to enhance linkage of genotype with phenotype, such metabolite-based genome-wide association analysis (mGWAS) for functional genomics (Adamski and Suhre 2013; Luo 2015).

A pilot study has been undertaken to investigate utility of metabolomics within yam breeding programs. Metabolite profiling a set of *Dioscorea* accessions routinely used in yam breeding programmes has been illustrated. Such approaches will facilitate the capture of biochemical diversity within this crop species, enable comparisons across other tuber food crops and contribute to the deciphering of underlying biochemical and molecular mechanisms associated with agronomic and consumer traits.

A GC-MS platform was opted for as costs are not as prohibitive for developing countries when compared to LC-MS, NMR and other approaches. The relatively reduced complexity of data analysis, ease of operation and higher reproducibility would hopefully facilitate skill transferability, education and capacity building enabling incorporation of metabolomics in yam breeding programmes at national and regional levels.

## Results

**Fig. 1:**
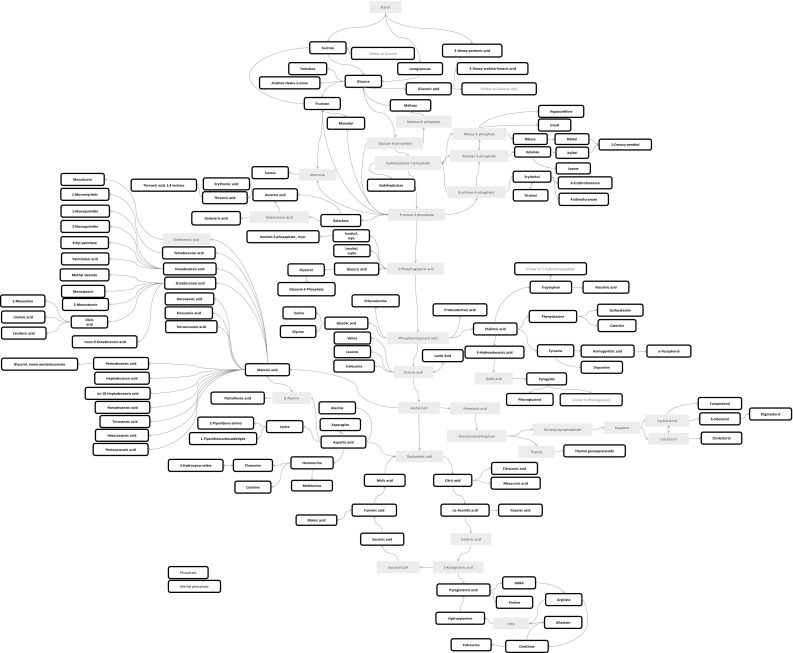
Map of measured primary metabolome of *Dioscorea* tubers. Visual representation of biochemical pathways of compound recorded in tuber extracts by the GC-MS profiling platform. Metabolites with preliminary identification are shown in green. Metabolites not detected by the platform are shown in red

Using a GC-MS based platform, tuber material from 49 accessions across four *Dioscorea* species from the global breeding program was analysed (Table 1). Collectively, 152 metabolites (Table S1) were identified based on the guidelines produced by the international metabolomics society (Fernie et al. 2011; Sumner et al. 2007). A customised *Dioscorea* GC-MS metabolite library, previously built using extracts from leaf tissue (Price et al. 2016), was extended to include non-polar metabolites providing comprehensive coverage of key sectors of *Dioscorea* metabolome found in tubers. Additionally, 89 unknown molecular features have also been measured (Table S2) to allow comprehensive platform coverage. Using these data, a model of the tuber biochemical pathways has been created (Fig. 1).

**Table 1:** *Dioscorea* accessions sourced from the IITA Yam Breeding Unit of the International Institute of Tropical Agriculture

*Dioscorea* species [in text]	Abbreviation	Accession Numbers
*alata* L. [*D. alata*]	TDa	98/01176
		297
		98/001166
		291
		00/00194
*bulbifera* L. [*D. bulbifera*]^a^	TDb	3059
		3079
		3072
		3688
		3048
*cayennensis* Lam. subsp. *cayennensis* [*D. cayennensis]*	TDc	04-71-2
		03–5
		95 − 17
		04-97-4
*dumetorum* (Kunth) Pax [*D. dumetorum*]	TDd	08-38-8
		08-36-14
		3108
		08-14-42
		1315
		3112
		4118
		4088
		08-37-12
		3774
		08-37-27
		08-37-16
		3104
		08-38-57
		3947
		05–6
		3100
		08-36-12
		08-38-18
		3109
		3648
		08-14-6
		08-36-88
		08-13-1
		08-3879
*cayennensis* Lam. subsp. *rotundata* (Poir.) J. Miège [*D. rotundata*]	TDr	EHObia
		99/02607
		omi-Efun
		97/00917
		95/01932
		97/00793
		97/00777
		04-219
		EHuRu
		Ponna

^a^Aerial tubers (bulbils) were provided for accessions of *D. bulbifera*

Of the 152 identified compounds, 41 were not detected in one or more species, with 9 being unique to an individual species and a further 7 uniquely absent in only one species. However, these differences were accession specific and it was not possible to classify species based on presence/absence profiles. Twenty-eight of the identified compounds were detectable in both polar and non-polar extracts (Table S1), which is not unexpected given the crude nature of separation between phases. Multivariate statistical analysis was conducted on both polar and non-polar extracts independently along with the combined data set.

**Fig. 2:**
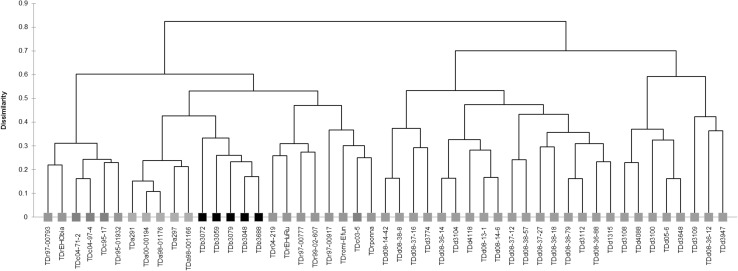
Dendrogram of *Dioscorea* accessions using identified compounds measured by the GC-MS platform. Complete-linkage agglomerative hierarchical clustering (AHC) on the Spearman dissimilarity matrix of mean (n = 3) metabolite abundances recorded in tuber extracts. *Dioscorea dumetorum* (blue); *D. rotundata* (green); *D. alata* (yellow); *D. cayennensis* (red) and *D. bulbifera* (black) recorded in triplicate

**Fig. 3:**
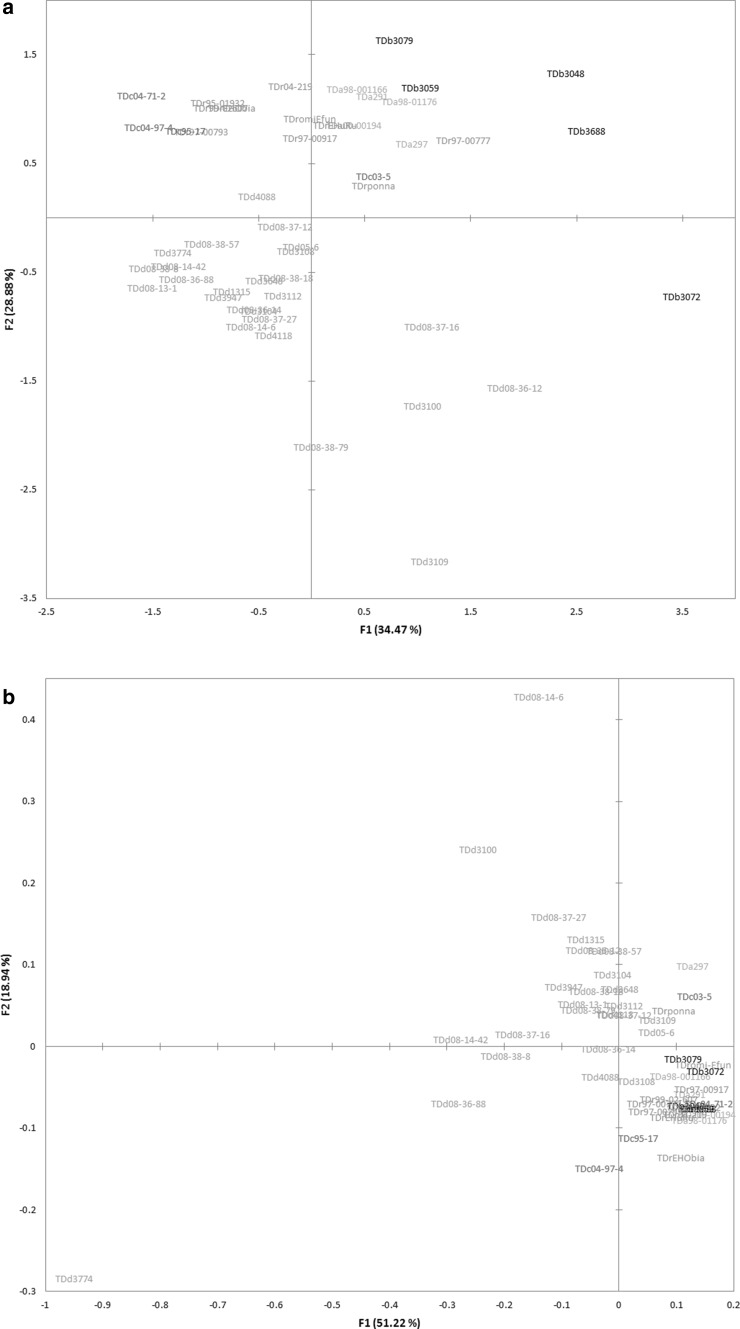
GPA of **a** polar and **b** non-polar extracts from tuber material of *Dioscorea* accessions. Consensus (n = 3) configurations following Generalised Procrustes Analysis conducted on all metabolite features recorded in the respective phases. *Dioscorea dumetorum* (blue); *D. rotundata* (green); *D. alata* (yellow); *D. cayennensis* (red) and *D. bulbifera* (black) recorded in triplicate (n = 3)

Clustering on the combined dataset showed that total metabolite profiles and the composition of identified metabolites provide the same separation of material (Figure S1). Species were largely distinguishable, though variants of *D. rotundata* and *D. cayennensis* showed overlap and grouped together (Fig. 2). Discrimination among species was also apparent when profiling polar extracts (Fig. 3a), with separation along principal component (PC) 1 driven by sugars and that on PC2 via organic acids, yet not possible following analysis on the non-polar dataset (Fig. 3b). Notably, accession *TDd 3774* (*D. dumetorum*) largely diverged from the sample set due to an abundance of fatty acids (Figure S2) however, presented a polar profile indiscriminate from many other accessions of the *D. dumetorum* (Fig. 3a). Excluding *TDd 3774* showed that within non-polar profiles two groups were apparent: those possessing an abundance of sterols and fatty acids versus those with low amounts (Figure S3).

**Fig. 4:**
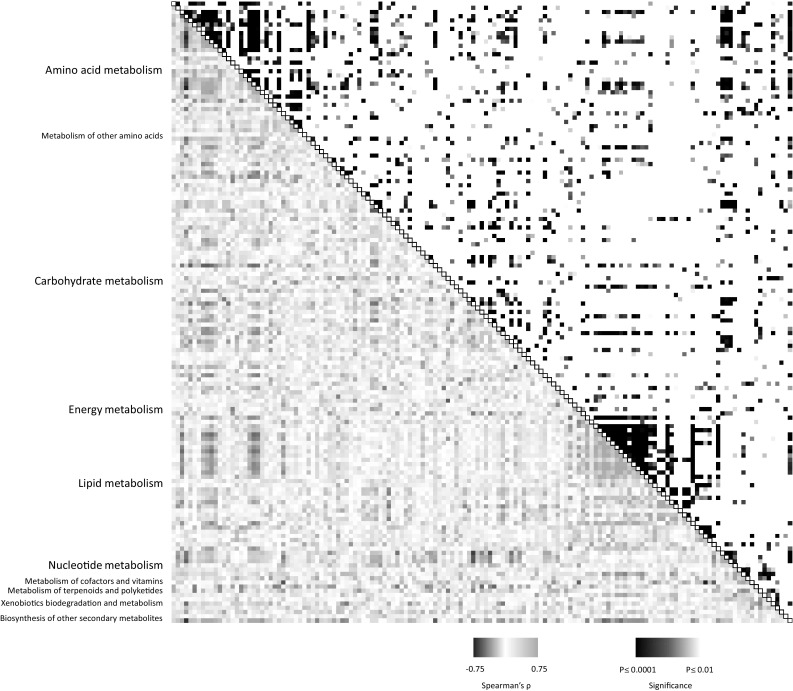
Heat-map of metabolite–metabolite correlation for *D. dumetorum*. Spearman correlation between metabolites across all replicates of *D. dumetorum* (25 accessions, n = 3) shows that compounds typically have significant correlations within compound class and among biochemically-related pathways. In the coloured area rectangles represent Spearman’s rho and in the black and white area, rectangles represent the respective p-value. Individual metabolites shown in Figure S4

Representation of the abundance of the most discriminatory variables in the most diverse accessions highlighted complex distributions of metabolites across accessions (Figure S4). As per qualitative differences, quantitative differences were largely accession specific. Metabolite–metabolite correlation on *D. dumetorum* accessions showed that many biochemically-related compounds correlate (Fig. 4, Figure S5), e.g. amino acids positively correlated within their own class and with nucleotides and cofactors and secondary metabolites and fatty acids positively correlate with their own compound class whilst fatty acid derivatives negatively correlate with other fatty acids.

The platform can provide robust analysis relevant to underlying metabolic pathways and thus has potential to investigate metabolic perturbation due to different biotic and abiotic stresses or investigate postharvest physiological deterioration. For example, the accessions of *D. dumetorum* could be segregated into three lineages (Figure S6). Class 1 (red branch) showed increased levels of amino acids and phosphate, Class 2 (green branch) were distinguished by glycerol mono-pentadecanoate and homogentisic acid whilst Class 3 (blue branch) showed increased abundances of sugars and fatty acids.

### Analysis of metabolite gradients over tuber sub-sections

Different sections of yam tuber show different enzymatic activities (Oluoha 1988; Wellington and Ahmad 1993; Wheatley et al. 2002) and phenotypic differences reportedly observed when mini-sett sections are used as planting material. To investigate the impact on metabolic functions a representative accession was selected per each species and analyses conducted on the head, middle, tail and skin sections of tuber independently.

In total, 162 metabolite features were detected and 93 identified (Table S3). The skin from all tubers displayed a similar profile, distinguished from other sections of tuber largely due to reduced amino acid content (Figure S7). Only a limited number of metabolites showed significant differences across different tuber sections; however these were species-specific with no single metabolite showing significant differences across sections for all species (Figure S8). All tubers, except those of *D. rotundata*, showed a gradient of malic acid concentration from highest abundance in tail to lowest in head. *Dioscorea bulbifera* showed a low-to-high gradient for many amino acids from head-to-tail and the same gradient was evident in *D. dumetorum* but for sugars. Sections of *D. rotundata* showed the highest number of compounds which were significantly different in Sections (16) however; there were not clear distribution patterns or metabolite gradients evident for many compounds. Despite different enzymatic activity having been shown from different portions of yam, these previous studies could not define patterns of biochemical properties across head, middle and tail sections. Additionally, the results were not unexpected as work across sections of potato noted that large differences between individual tubers, plants and growing season limited interpretation (Merlo et al. 1993).

### Tuber and leaf comparison

**Fig. 5:**
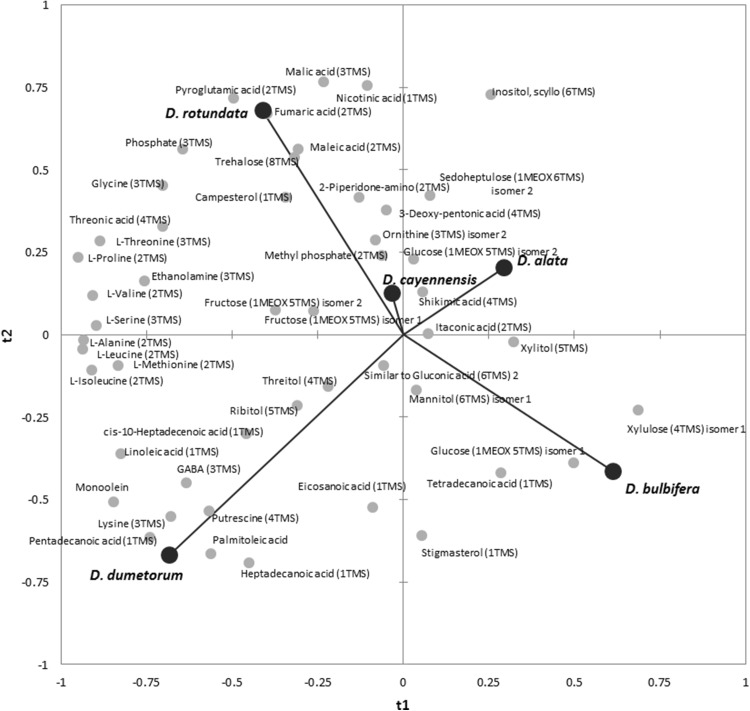
A reduced PLS-DA model classifies species based on leaf and tuber metabolite profiles. Loading plot of metabolites recorded in leaf and tuber extracts used as variables to predict species. The top 50 variables of importance (VIPs) were selected from an initial PLS-DA model created using all identified metabolites and validated using a random subset of > 25% of variables recorded (Figure S9)

Using the method developed, a second crop at a different geographical location was produced to provide a comparison between tuber and leaf material. In this case the material was generated under polytunnel conditions in the UK. In total 337 metabolite features were measured via the platform, 258 in tuber and 312 in leaf respectively (Table S4). Preliminary analysis showed that similar pattern of species separation was evident for tuber and leaf material (Figure S9) and so a PLS-DA model using species as classifier was created (Figure S10). The model allowed species classification (90% accurate on validation set) with only a sample of *D. cayennensis* wrongly predicted as being *D. rotundata*. The top 50 variables of importance in the model were selected and used in a new model to distinguish metabolites driving species delineation (Fig. 5). *Dioscorea dumetorum* was defined by fatty acids, *D. rotundata* and *D. cayennensis* by TCA cycle intermediates and phosphate, and *D. alata* and *D. bublifera* both largely by sugars.

## Conclusions

A diverse panel of accessions from four *Dioscorea* species routinely used in yam breeding programmes was used to create a semi-automated GC-MS library for chemo-typing. The library provides comprehensive coverage of intermediary metabolism and abundant specialised metabolites. A pathway map of the measured yam tuber metabolome was created (Fig. 1). *Dioscorea* tuber presents many challenges for metabolomics analysis due to high starch content yet, simple modifications to conventional methods (such as an extended centrifugation time and chilling of extracts prior to evaporation) allowed robust and repeatable metabolite profiling.

Exploratory analysis of phenolic compounds (Champagne et al. 2011) and carotenoids (Champagne et al. 2010) in some *Dioscorea* species showed both inter- and intra- species diversity and emphasised the potential of global metabolomic investigation. Previous study evidenced that species could largely be discriminated by metabolite profiling polar extracts of leaf (Price et al. 2016), which has further been shown true for tuber material in this work (Fig. 3).

The chemotaxonomic relationship of species (Fig. 2) also closely matches that of phylogenetic studies, with *D. dumetorum* being most divergent and monophyletic support for *D. bulbifera* and *D. alata*, whilst *D. rotundata* and *D. cayennensis* are more genetically similar (Chaïr et al. 2005; Sartie et al. 2012). Other studies distinguished *D. rotundata* from *D. cayennensis* using molecular markers (Ramser et al. 1997), morphological characteristics and isozyme markers (Dansi et al. 2000; Hamon and Touré 1990) with discriminatory enzymatic descriptors related to central carbon metabolism of leaf. Whilst the measured tuber metabolome did not discriminate between the two cultigens (Fig. 2), inclusion of leaf material may offer increased resolution, as evidenced in the exploratory study conducted (Fig. 3).

Following global metabolomics approaches, redundancy within the biochemical profiles of these breeding materials concealed trends (Figure S1) and limited data interpretation. This is a common bottleneck for analysis of metabolomics investigations (Kuehne et al. 2015). However, detailed analyses reveal variation across broad pathways in an accession-specific manner (Figure S4). Metabolite profiling of both tuber and leaf material revealed that the same trends could be observed in leaf as tuber material (Figure S9) and though a small set of accessions was studied, a sub-selection of metabolites could be used to accurately predict species (Fig. 5, Figure S10). Similar trends have been observed in other tuberous crops such as cassava, potato and sweet potato. Future analysis using bigger datasets will be needed to validate if the leaf profiles can be representative or even predictive of tuber metabolite composition. If so, initial phenotypic screening of breeding programs could be undertaken on leaf material and profiling of root and tuber crops would be significantly more rapid; conducted prior to tuber formation; requiring less labour for material harvesting and analyses being cheaper and easier.

Identifying biochemical signatures of phenotypic traits would allow metabolite-marker based breeding (Fernie and Schauer 2009). Given the lack of genomic information and genetic resources that currently hinders the applicability of gene based marker-assisted breeding (Bhattacharjee et al. 2011; Hana; Chaïr et al. 2016; White et al. 2016), implementing metabolomics offers great potential for near-future crop gains. Metabolite profiles provide a direct biochemical measurement of quality traits which can be utilised as markers to investigate trait inheritance independent of association with genetic mechanisms. Additionally, information derived from integrating biochemical profiles with the morphological descriptors traditionally recorded throughout breeding programs can subsequently be integrated with genomic data when it becomes available, making the characterisation of genes much more rapid (Tohge and Fernie 2010). The genome sequence of *D. rotundata* has recently been published and sex-linked markers determined (Tamiru et al. 2017). However, transferability of genetic markers to other species is dependent on taxonomic closeness (Tamiru et al. 2015) and differing sex-determination mechanisms between species and environmental plasticity may still hinder breeding. In comparison, metabolite markers may be easier to implement in multi-species crop breeding programmes, such as for yam and are simpler to associate with environmental factors. Additionally, in wheat metabolite markers have been shown to be as equally predictive of complex traits as genetic markers (Riedelsheimer et al. 2012) and more effective when use of both is combined (Ward et al. 2015). Thus, integrating a metabolomics component into current genetics-led yam breeding would hopefully remove some of the constraints realised for typical breeding approaches.

In 2014, Adaramola et al. (2014) integrated ploidy, qualitative phytochemical content and morphological traits to design *D. dumetorum* breeding strategy; however, phytochemical analyses were crude and only a weak association between terpenoid detection and ploidy was found. The relationship between gene dosage and quality traits is complex, with many levels of regulation (transcriptional, translational, enzymatic etc.) and genotype to phenotype relationships indirect, especially in mixed polyploid crops such as yam. Biochemical markers are more closely associated to phenotypic traits and dynamic measurements of metabolome composition are able to represent phenotypic changes. Therefore, metabolome to phenotype relationships can provide a simplified system with which to assess quality traits and provide a stepping stone for interpretation of genotype-phenotype interaction.

Within the limits of the platforms dynamic range it was clear that the total number of metabolites detected in tuber was less than those found in the leaf. Typically the differences were both quantitative and qualitative, with fewer pathway intermediates such as sugar derivatives and a reduced number of phenylpropanoid pathway derived compounds. This is to be expected, given the abundance of sucrose reduces the dynamic range measurable and lower expression of phenylpropanoid metabolism in tuber. Information on post-harvest storage of root and tuber crops is important for these types of study, with far more metabolomics signatures recorded when the crop is immediately harvested, allowing easier differentiation among species etc. Time-series studies on each species could be undertaken in future studies to investigate crucial aspect of post-harvest deterioration.

Moreover, alternative platforms such as LC- or CE-MS and NMR could be used and would provide enhanced coverage of the metabolome and increase accession demarcation. In particular, enhanced measurement of secondary metabolites would aid more detailed analysis and is advocated when investigating complex quality traits such as disease susceptibility, tolerance and resistance and sensory-related parameters.

This study showcases comprehensive practical analysis of the yam metabolome. Application of this technology to a larger sample set of the breeding program is needed with integration of data from multiple ‘–omics’ platforms to provide a broader systems level knowledge base. Since metabolomics only provides a temporal snapshot of biochemical phenotype, analysis over numerous growing seasons will be required to identify robust quality trait markers. However, being able to correlate biochemical signatures with numerous agronomic and sensorial traits and investigate inheritance independent of the underlying genetic mechanisms provides potential to speed breeding increasing the rapidity of selection. Interpretation of data from the platform is not compromised by the complicated genetic architecture which slows genomics approaches and in turn provides a segue for future elucidation and association of genotype to phenotype.

This work increases the phytochemical resources available for yam and will facilitate the deciphering of underlying molecular and biochemical mechanisms associated with traits of interest; add value to the discovery pipeline and provide a resource to aid rational design of breeding towards better varieties that are suitable for future sustainable intensification.

## Methods

### Reagents

All reagents were of analytical grade.

### Plant material

Forty-nine accessions covering four species of *Dioscorea* were sourced from the IITA Yam Breeding Unit of the International Institute of Tropical Agriculture (IITA), Ibadan, Nigeria (Table 1) and shipped to the Royal Holloway University of London (RHUL), United Kingdom for further analysis.

For standard extractions tuber from three biological replicates per accession were sectioned laterally and longitudinally into 12; and six representative sections per tuber frozen in liquid nitrogen (Figure S10). Sections were freeze-dried; skin peeled and ground (via a cryogenic mill) to a homogenous powder prior to extraction. All samples were stored at − 80 °C prior to further processing.

Five accessions (*TDa 98-01176, TDb 3059, TDc 04-71-2, TDd 08-14-42, TDr EHuRu*) underwent spatial metabolomics analyses whereby tuber from a fourth biological replicate was sectioned into individual head, middle and tail portions (Figure S10). Each section was then processed as above and analysed with triplicate technical repetition. The peeled skin was also analysed in duplicate.

Additionally, tuber of a fifth replicate for these accessions was divided into mini-setts planted in polytunnel at RHUL, UK. Tuber and leaf material from three plants per accession were harvested following 9 months growth (May 2014–January 2015). Tuber material was processed as per standard extracts, yet on the same day as harvest. Leaf material was processed as previously described (Price et al. 2016).

### Gas Chromatography-Mass Spectrometry (GC-MS) Profiling

Material was extracted, derivatised and analysed via GC-MS as previously described (Price et al. 2016) with modifications: complete phase separation of MeOH:H_2_O/CHCl_3_ required centrifugation (14,000 rpm) for 10 min. Polar phase aliquots were stored overnight at − 20 °C and then dried under inert nitrogen. Samples were subsequently stored at − 80 °C. Both polar and non-polar aliquots were analysed; except portions of head, middle and tail, where only polar aliquots were analysed. Only splitless injections onto the GC were undertaken. All matches to NIST database were of a probability greater than 80%. Peaks were only included in analyses if identified in all three replicates of any sample for each study.

### Data processing and statistics

Polar and non-polar datasets were combined; removing duplicates by selecting the maximal response recorded per each compounds present in both phases.

All data analyses were performed using XLSTAT add-ins (Addinsoft) within Microsoft Excel. Generalised Procrustes Analysis (GPA) was performed using the Commandeur algorithm with 300 simulations. Samples which were more visually divergent on the GPA plots were selected for representation in scattergrams. Agglomerative hierarchical clustering (AHC) was performed using complete linkage on the spearman dissimilarity matrix. Partial Least Squares Discriminant Analysis (PLS-DA) was performed on centred and reduced variables and validated by a random subset of ≥ 25% of variables. Metabolite–metabolite correlations were performed via spearman correlation coefficients and heat-maps arranged via assignment of compounds into KEGG pathways. Pathway displays were created in Microsoft Powerpoint. Kruskal–Wallis’ one way analysis of variance were performed following normality and variance testing. Monte Carlo permutations (10,000) were used for p-value calculation. Conover–Iman post hoc tests (α = 0.05) were Bonferroni- corrected and the selection of most discriminatory metabolites based on the number of groups generated i.e. the more groups a metabolite segregated the accessions into, the more discriminatory the metabolite. All univariate tests were two-tailed.

### Accession numbers

Provided in Table 1.

## Electronic supplementary material

Below is the link to the electronic supplementary material.

Supplementary material 1 (PDF 2806 KB)

ST1. Mean abundance of identified metabolites recorded in tuber extracts of *Dioscorea* accessions following standard extractions (XLSX 118 KB)

ST2. Unidentified metabolite features recorded in tuber extracts of *Dioscorea* accessions. (XLSX 12 KB)

ST3. Mean abundance of identified metabolites recorded in head, middle and tail sections of tuber. (XLSX 38 KB)

ST4. Mean abundance of metabolites recorded in leaf and tuber extracts of accessions grown at RHUL. (XLSX 61 KB)
