# Supplementary material for: Metabolite profiling of yam (Dioscorea spp.) accessions for use in crop improvement programmes
Source: Metabolomics. 2017 Oct 14;13(11):144. doi: 10.1007/s11306-017-1279-7 (PMC5641283; doi:10.1007/s11306-017-1279-7)
Supplement: Supplementary file 1 — Supplementary material 1 (PDF 2806 KB) [file 11306_2017_1279_MOESM1_ESM.pdf]

Figure SF1

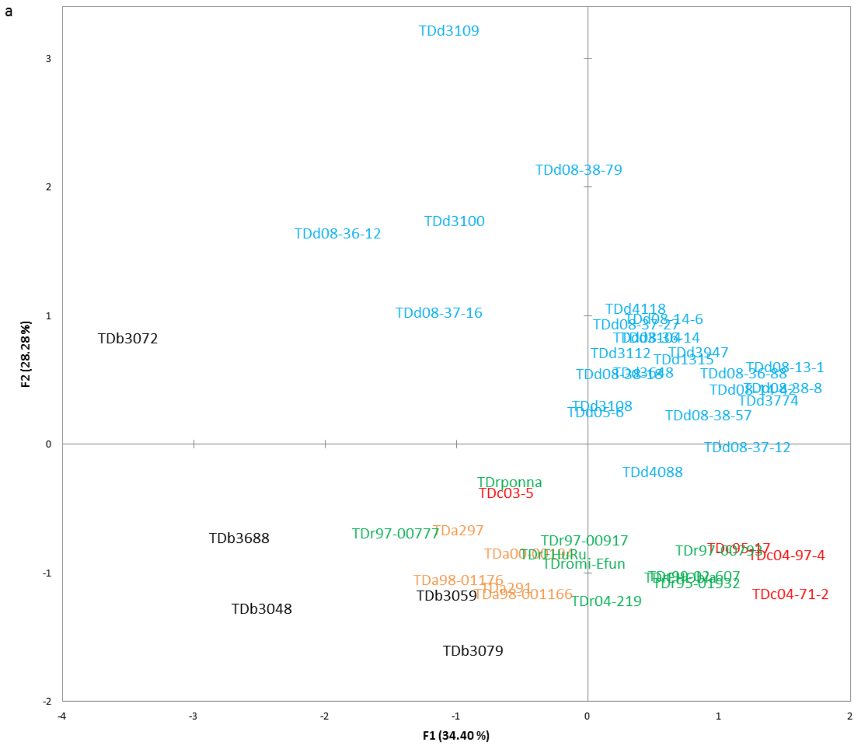

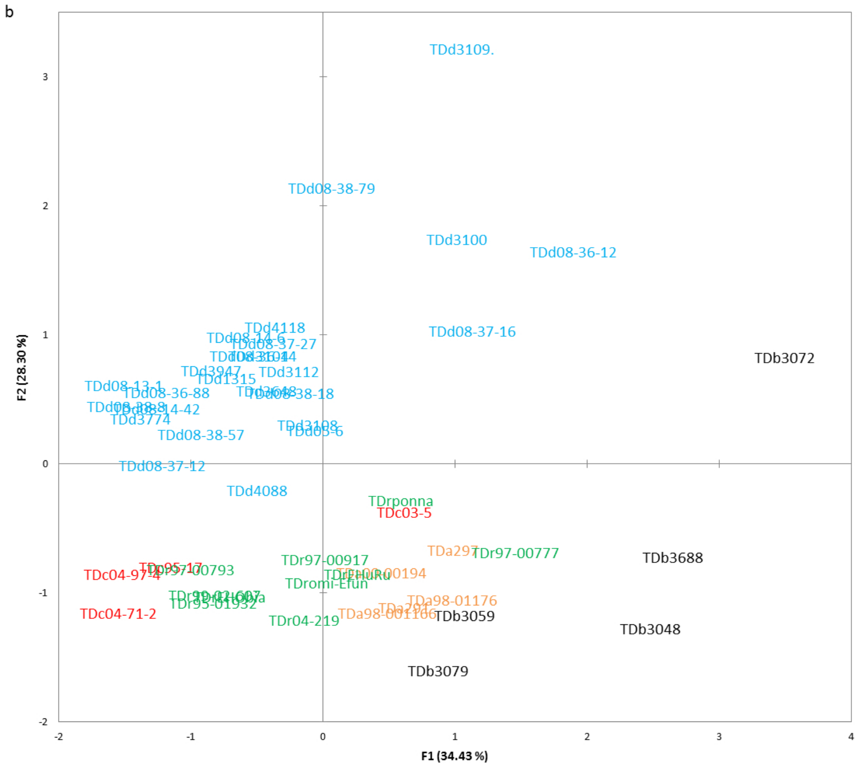

SF1. GPA bi-plots following GC-MS analysis on tuber extracts. Consensus (n=3) arrangements using (a) all metabolite features and (b) only identified metabolites shows the same species and sample discrimination. D. dumetorum (blue); D. rotundata (green); D. alata (yellow); D. cayennensis (red) and D. bulbifera (black).

Figure SF2

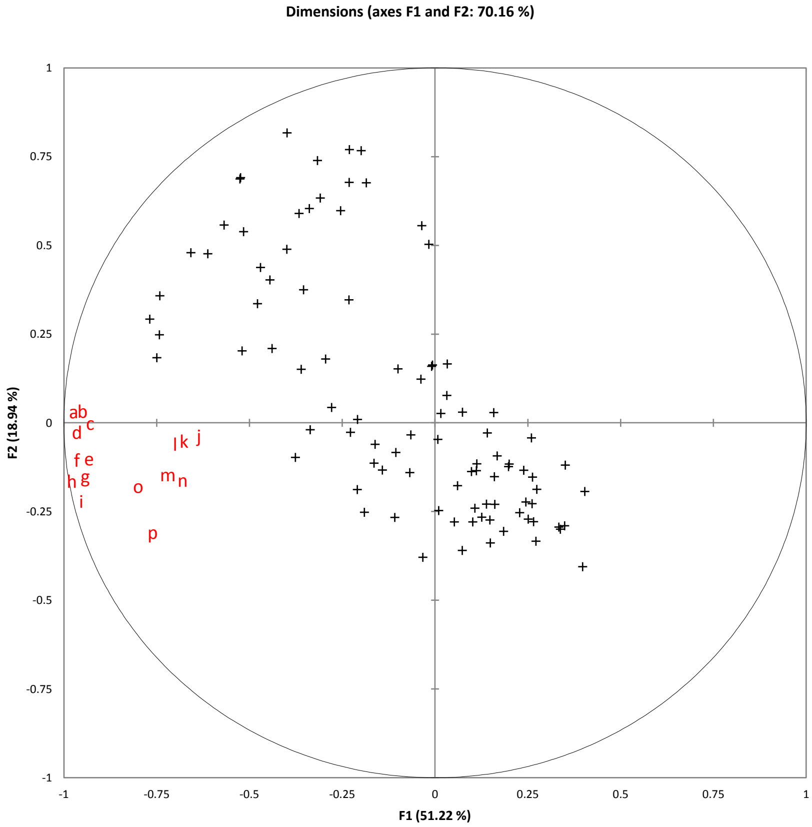

SF2. Loading plot of GPA following GC-MS analysis on non-polar extracts of tuber. Score plot shows metabolite (+) loadings for Figure 3b. Red letters represent the metabolites which cause promote diverge of accession TDd3774: a, Pentadecanoic acid (1TMS); b, Eicosanoic acid (1TMS); c, cis-10-Heptadecenoic acid (1TMS); d, Palmitoleic acid (1TMS); e, Nonadecanoic acid (1TMS); f, Heptadecanoic acid (1TMS); g, a-Linolenic acid (1TMS); h, Linoleic acid (1TMS); I, Hexadecanoic acid (1TMS); j, Hexadecanoic acid (1TMS); k, Unknown; l, Linoleic acid, methyl ester (1TMS); m, Glycerol-3-Phosphate (4TMS); n, Octadecanoic acid (1TMS); o, Elaidic acid (1TMS); p, Oleic acid (1TMS).

Figure SF3

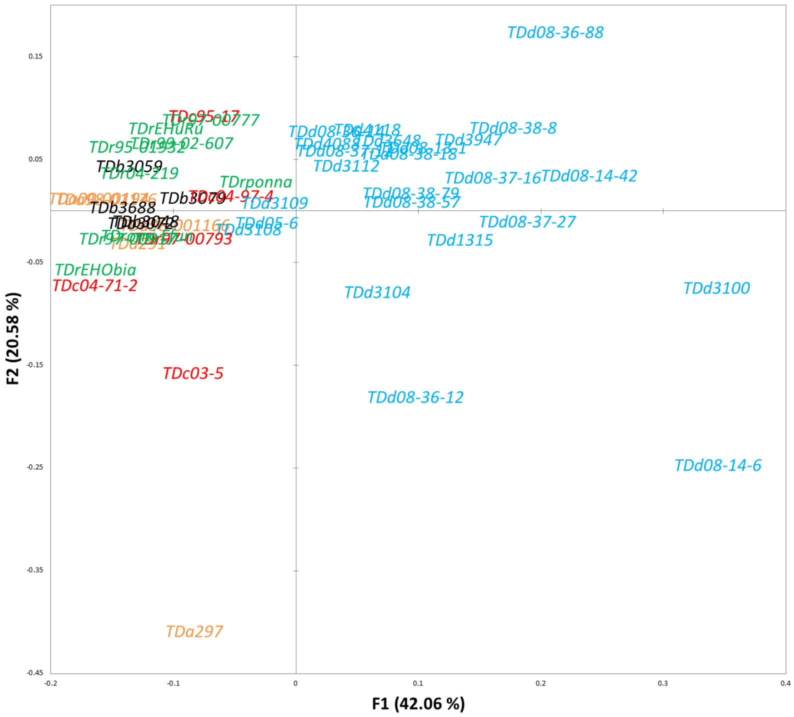

SF3. Consensus (n=3) GPA bi-plot of non-polar extracts from tuber excluding TDd 3774. All metabolite features detected in non-polar extracts following GC-MS analysis were included in the GPA. D. dumetorum (blue); D. rotundata (green); D. alata (yellow); D. cayennensis (red) and D. bulbifera (black).

Figure SF4

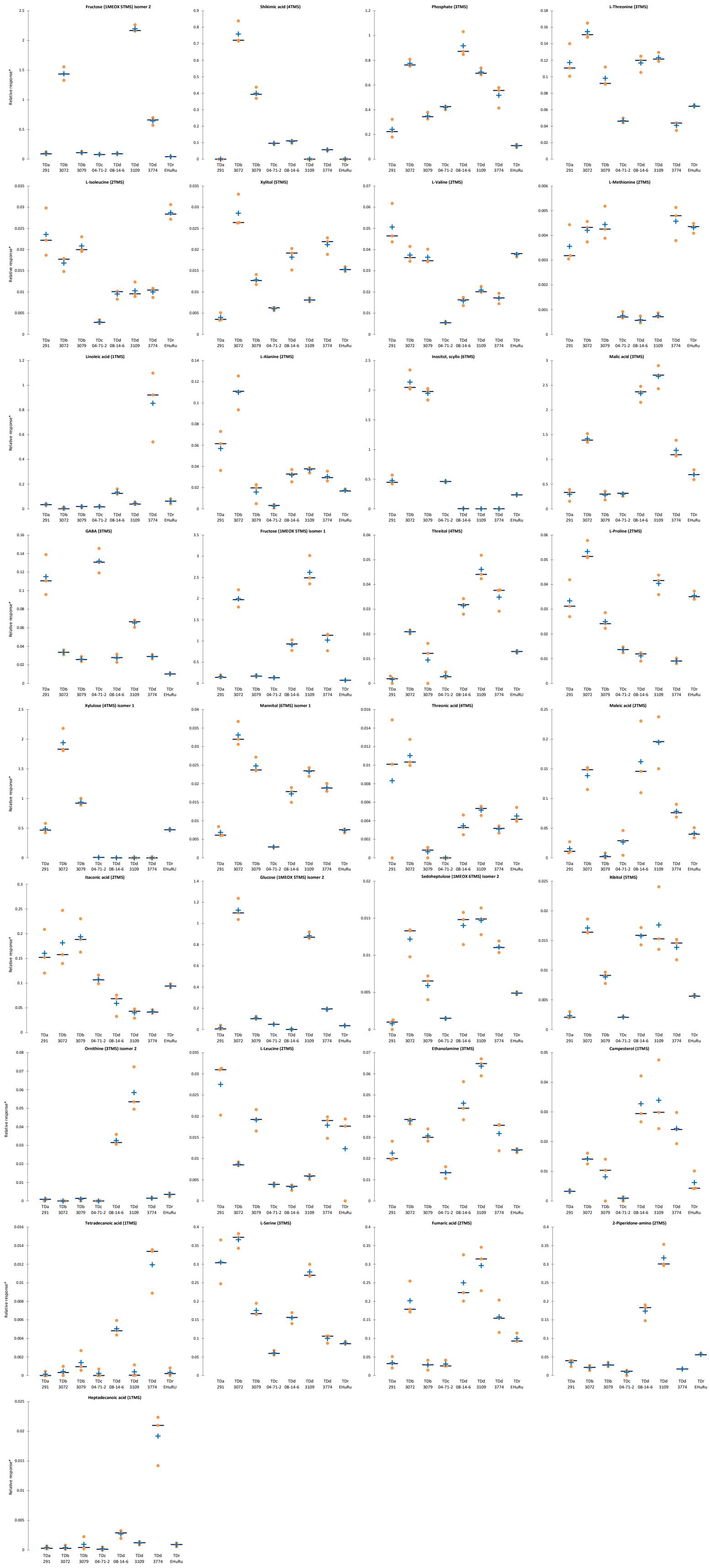

SF4. Scattergrams showing the abundance of compounds which are most discriminatory for tuber extracts. Mean (+), median (-) and individual measurements per sample (o) (n=3). Accessions were those visually most divergent on the GPA plots of polar and non-polar phase extracts (Figure 2). Abundance expressed as response relative to internal standard. Most discriminatory compounds selected based on number of groups generated by Bonferroni-corrected (α≤ 0.05) Conover-Iman post-hoc following Kruskal-Wallis one way analysis of variance on all 49 lines.

Figure SF5

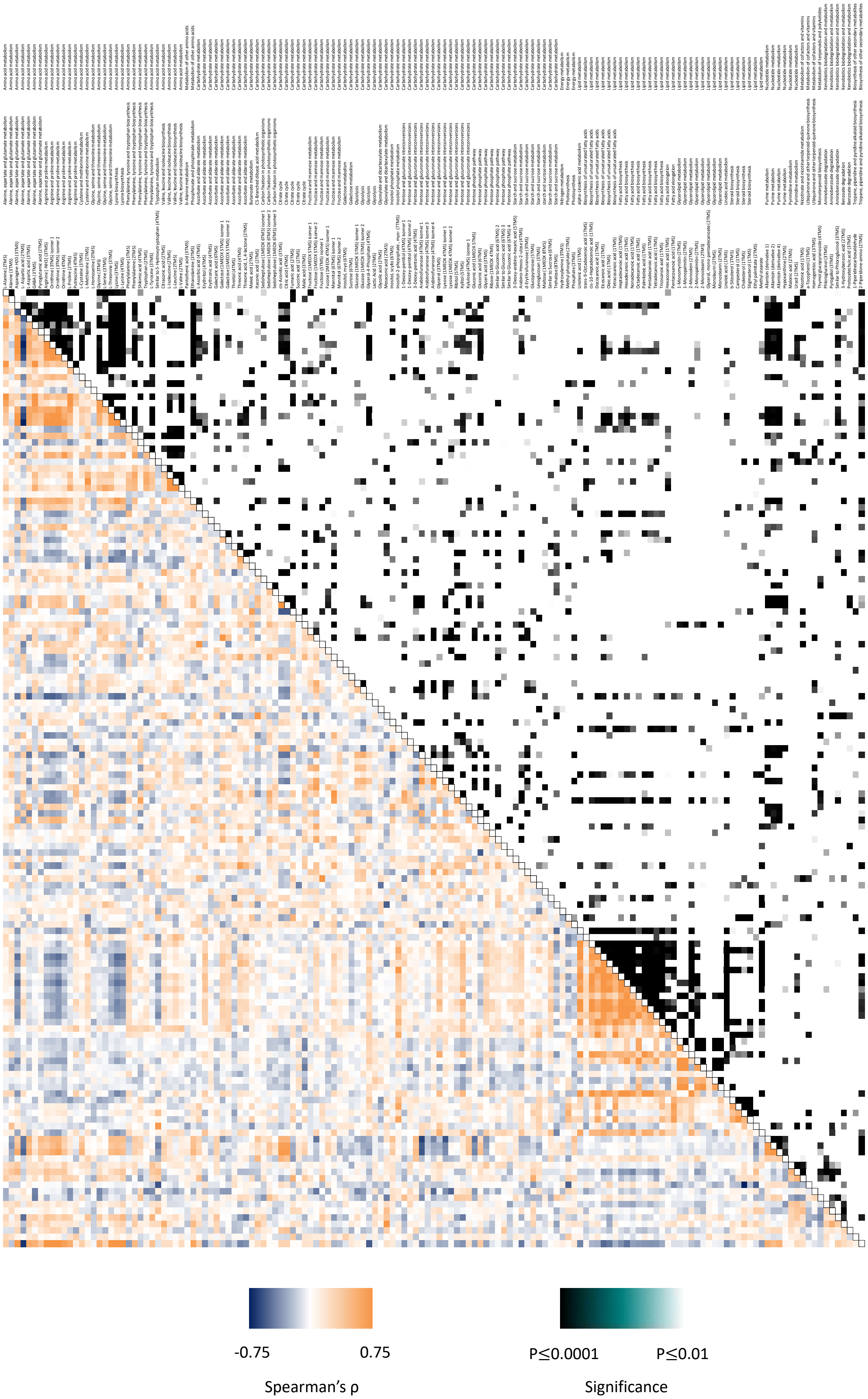

SF5. Detailed heat-map of metabolite-metabolite correlation analysis on D. dumetorum accessions. Spearman correlation between metabolites across D. dumetorum (25 accessions, n=3). In the coloured area rectangles represent Spearman's rho and in the black and white area, rectangles represent the respective p-value. Compounds have been put into compound classes based on KEGG Pathway assignments.

Figure SF6

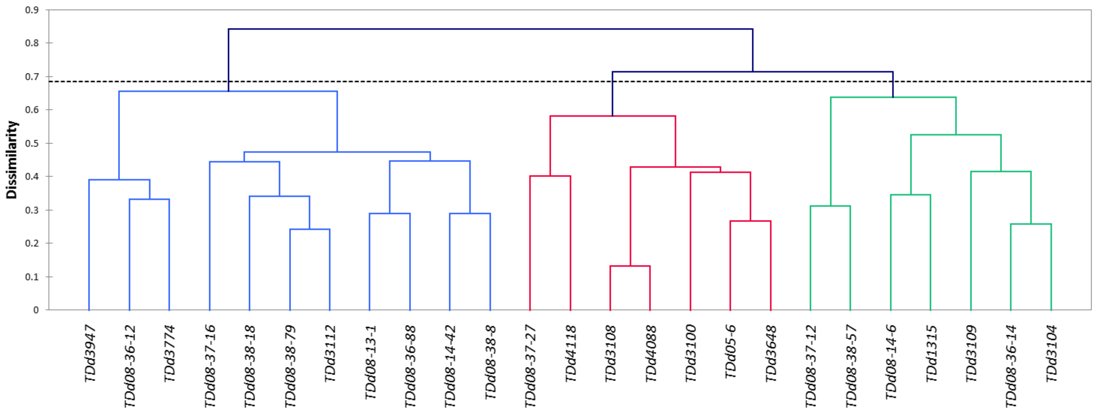

SF6. Clustering of D. dumetorum accessions using discriminatory metabolites. Complete linkage clustering on the Spearman dissimilarity matrix of mean (n=3) metabolite abundances of discriminatory metabolites distinguished the D. dumetorum accessions into three groups: Class 1 (red), Class 2 (green) and Class 3 (blue). Discriminatory metabolites were identified following Conover-Iman post-hoc following Bonferonni-corrected Kuskal-Wallis' analysis of variance testing.

Figure SF7

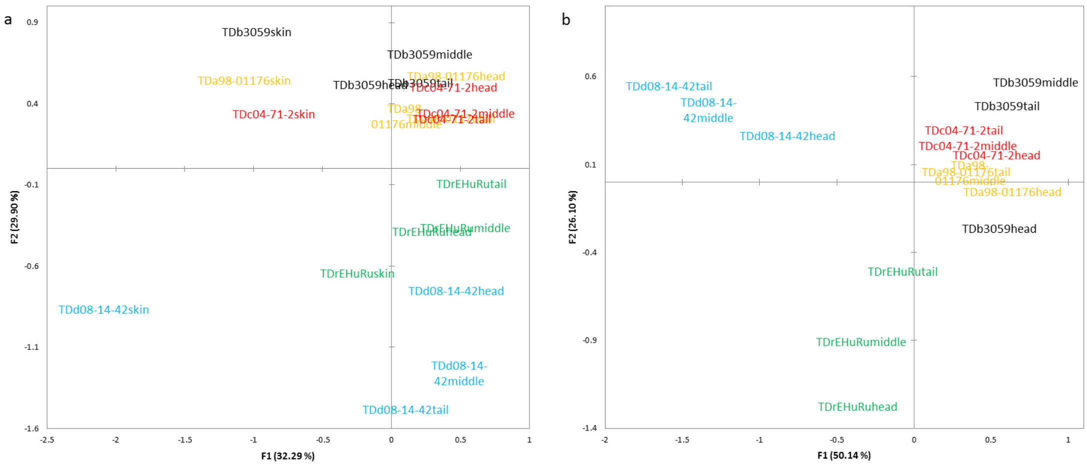

SF7. GPA analyses on polar extracts of individual sections of tuber. Tubers were sectioned into head (n=3), middle (n=3), tail (n=3) and skin (n=2) portions. (a) Consensus arrangement including the skin showed that skin portions were qualitatively different to other sections for all species. (b) Consensus arrangement excluding of the skin suggests compositional gradients are present across tubers. D. dumetorum (blue); D. rotundata (green); D. alata (yellow); D. cayennensis (red) and D. bulbifera (black).

Figure SF8

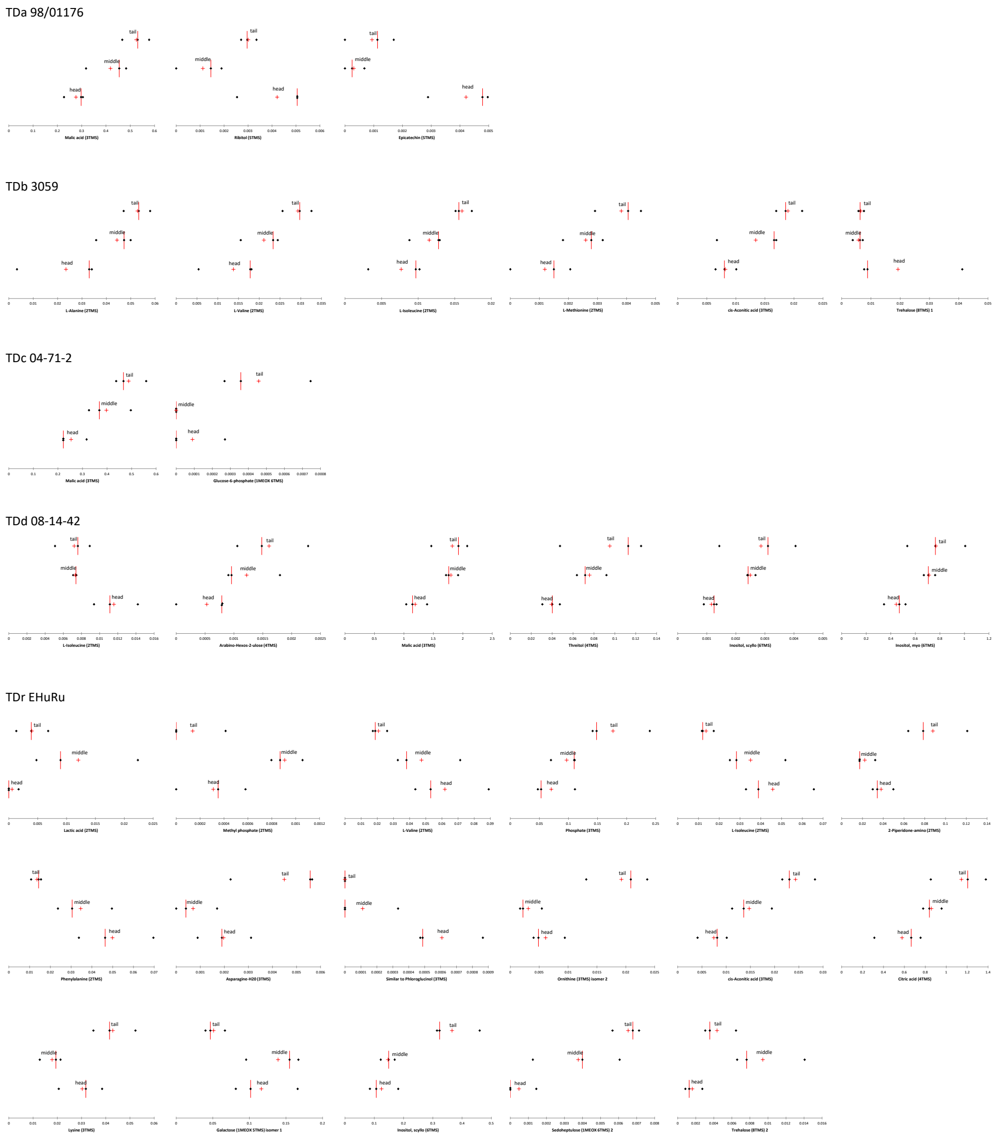

SF8. Scattergrams of metabolite abundance across different tuber sections. Visualisation of metabolites with significant (α ≤0.05) differences in abundance between head, middle and tail sections of tuber following Kruskal-Wallis' analysis of variance on each species individually. Abundance measured relative to internal standard. Replicates (black diamond), median (red vertical line), mean (red cross).

Figure SF9

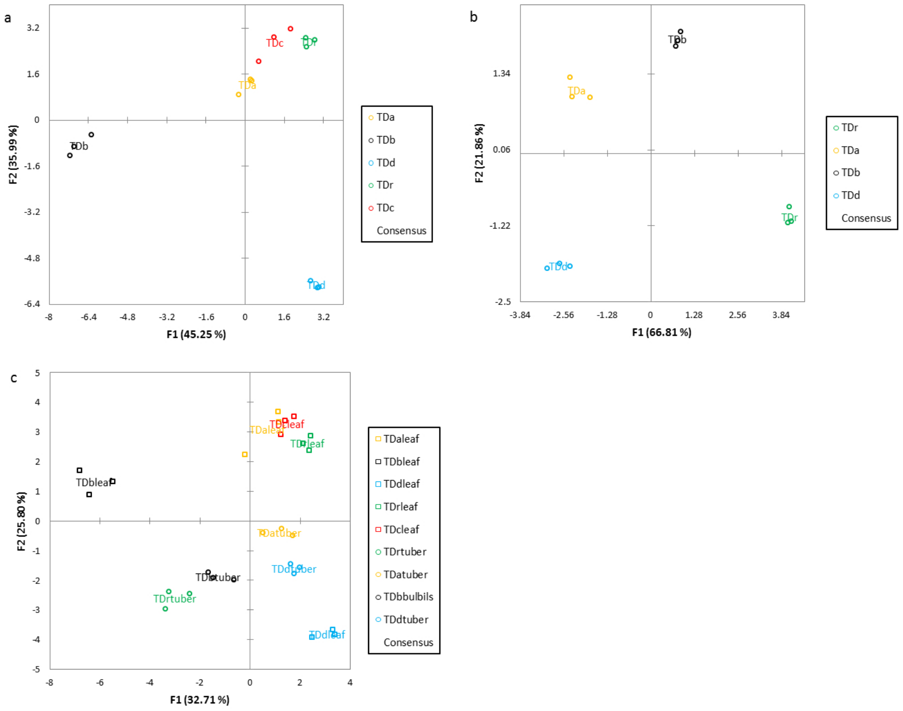

SF9. GPA analysis on the accessions grown in polytunnel. Analysis on (a) leaf and (b) tuber materials show that the same trend of separation is present, yet analysis on (c) the combined dataset does not allow easy interpretation. D. dumetorum (blue); D. rotundata (green); D. alata (yellow); D. cayennensis (red) and D. bulbifera (black). Leaf (circle), Tuber (square). GPA analysis included all metabolite features recorded in both polar and non-polar extracts via GC-MS performed in triplicate.

Figure SF10

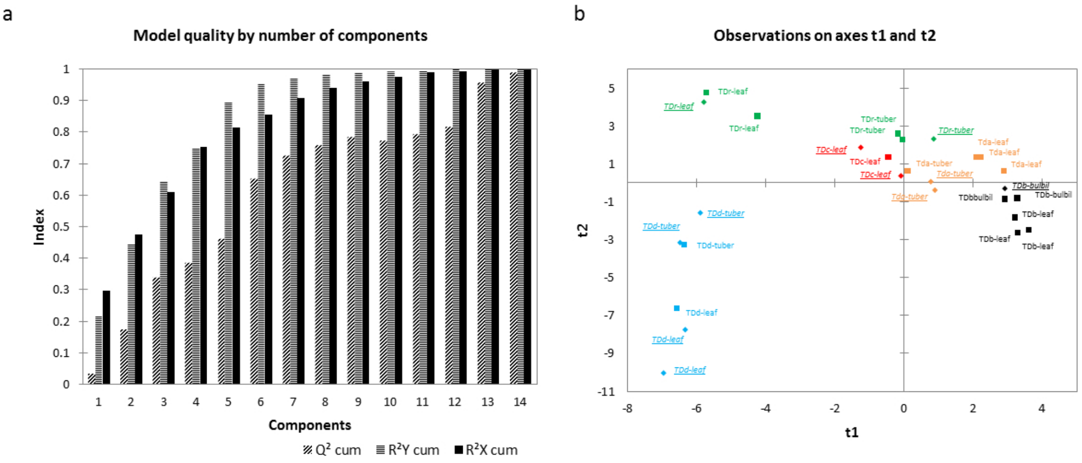

SF10. PLS-DA model using reduced metabolite profiles from tuber and leaf extracts. (a) Component indexes (a) and (b) observations bi-plot for a PLS-DA model built using the top 50 variables of importance (VIPs) from an initial PLS-DA model constructed using all identified metabolites. D. dumetorum (blue); D. rotundata (green); D. alata (yellow); D. cayennensis (red) and D. bulbifera (black).

Figure SF11

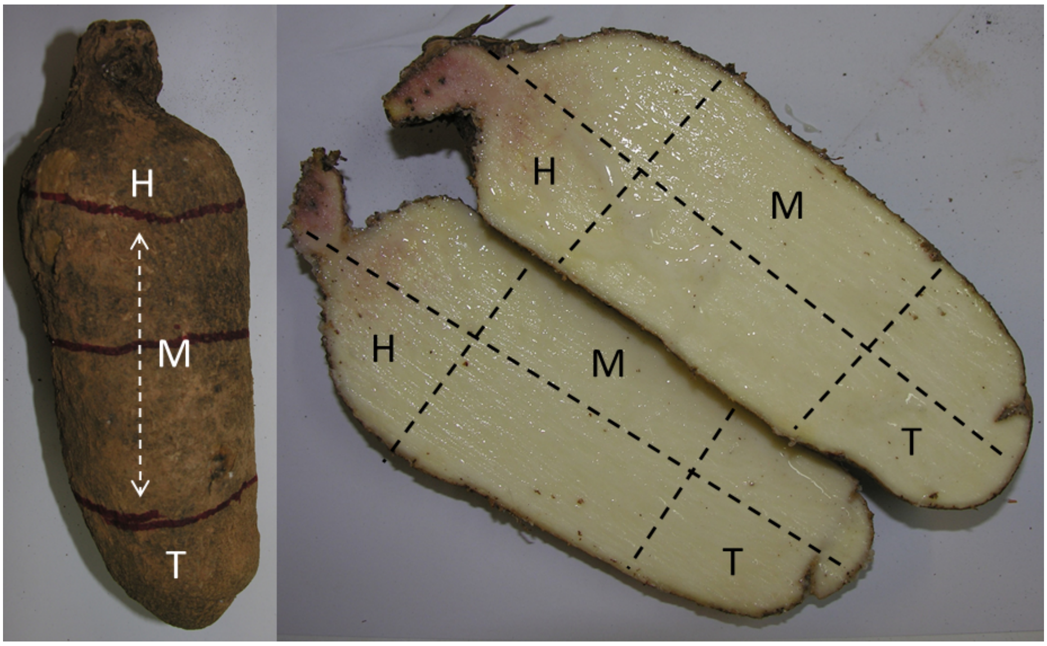

SF11. Sample preparation of tuber material. Sectioning of tubers into head (H), middle (M) and tail (T) portions for processing. For standard extractions H, M & T portions were pooled.
